# Near‐Infrared Spectrum of the First Excited State of Au_2_^+^

**DOI:** 10.1002/chem.202102542

**Published:** 2021-09-15

**Authors:** Marko Förstel, Kai Pollow, Taarna Studemund, Otto Dopfer

**Affiliations:** ^1^ Institut für Optik und Atomare Physik Technische Universität Berlin Hardenbergstr. 36 10623 Berlin Germany

**Keywords:** cations, electronic structure, gold, photodissociation, structure elucidation

## Abstract

Au_2_^+^ is a simple but crucial model system for understanding the diverse catalytic activity of gold. While the Au_2_^+^ ground state (X^2^Σ_g_^+^) is understood reasonably well from mass spectrometry and computations, no spectroscopic information is available for its first excited state (A^2^Σ_u_^+^). Herein, we present the vibrationally resolved electronic spectrum of this state for cold Ar‐tagged Au_2_^+^ cations. This exceptionally low‐lying and well isolated A^2^Σ_(u)_^+^←X^2^Σ_(g)_^+^ transition occurs in the near‐infrared range. The observed band origin (5738 cm^−1^, 1742.9 nm, 0.711 eV) and harmonic Au−Au and Au−Ar stretch frequencies (201 and 133 cm^−1^) agree surprisingly well with those predicted by standard time‐dependent density functional theory calculations. The linearly bonded Ar tag has little impact on either the geometric or electronic structure of Au_2_^+^, because the Au_2_^+^⋅⋅⋅Ar bond (∼0.4 eV) is much weaker than the Au−Au bond (∼2 eV). As a result of 6 s←5d excitation of an electron from the antibonding σ_u_^*^ orbital (HOMO‐1) into the bonding σ_g_ orbital (SOMO), the Au−Au bond contracts substantially (by 0.1 Å).

## Graphical abstract

**The Au_2_** ^**+**^ **cation**: The high‐resolution near‐infrared spectrum of the lowest excited electronic state of this simple diatomic cation recorded by Ar‐tagging photodissociation exhibits a surprisingly simple vibronic structure. Analysis by standard TD‐DFT calculations provides the first spectroscopic information about the chemical bond in Au_2_^+^ in both the ground and excited electronic states.

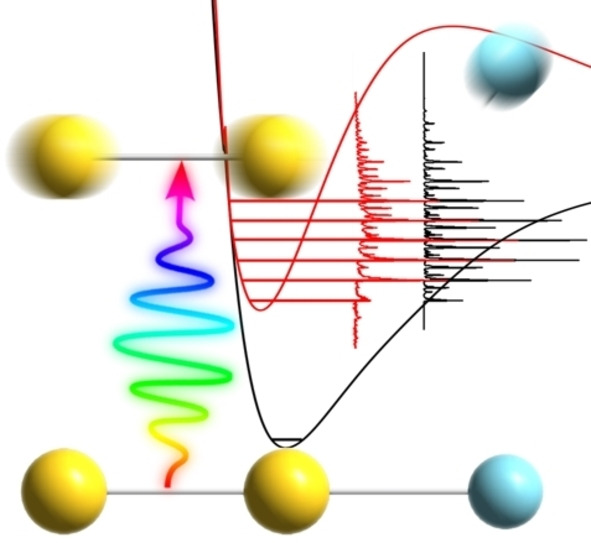

The often unusual chemical properties of small gold clusters arise from several factors, including strong spin−orbit coupling, contributions of d orbitals to chemical bonding, and large relativistic effects.^[1]^ The typical multi‐reference character of their excited electronic states, which are relevant for catalytic processes,^[2]^ provides high challenge for quantum chemical calculations, which are required to understand electronic structure and chemical reactivity at the molecular level.[^1^, ^3^] High‐resolution experimental spectra provide useful benchmarks for developing and testing such quantum chemical approaches.[^3^, ^4^] Recent progress in instrumentation has allowed our group to record for the first time vibrationally resolved electronic spectra of small and cold Au_*n*_^+^ cluster cations, such as Au_4_^+^ and Au_2_^+^, by means of photodissociation spectroscopy.[^3^, ^5^]

Concerning Au_2_^+^, we have so far characterized higher excited states in the 300–700 nm range, giving rise to two complex band systems near 440 and 325 nm, which both exhibit rather irregular vibronic structure due to strong coupling of multiple electronic states occurring in the same energy range.^[3a]^ This congested vibronic structure could only be explained by sophisticated multi‐reference calculations including spin–orbit coupling and relativistic corrections.^[3a]^ Clearly, standard time‐dependent density functional theory (TD‐DFT) calculations completely fail to reproduce the observed spectral pattern.^[3a]^ Due to its high binding energy (*D*_0_=2.2±0.2 eV),^[6]^ the lower electronic states of Au_2_^+^ cannot be probed by single‐photon dissociation from the cold ground electronic state. The lowest excited states of Au_*n*_^+^ clusters show a strong even‐odd alternation, and the open‐shell *n*=odd clusters have predicted transitions in the near‐infrared (NIR) range.^[7]^ In this respect, Au_10_^+^ exhibits a particularly low and broad transition centered at around 0.55 eV, which extends down into the vibrational domain of the ground electronic state. For Au_2_^+^, calculations predict an optically active and well isolated A^2^Σ_u_^+^ state around 0.8 eV above the X^2^Σ_g_^+^ ground state arising from 6 s←5d excitation of an electron from the antibonding σ_u_* orbital (HOMO‐1) into the bonding σ_g_(s) orbital (SOMO), as shown in Figure 1.[^3a^, ^6^] As no other (bright) states are nearby, coupling to other states is expected to be weak, at least near the potential minimum of the A state. As a result, the anticipated simpler vibronic spectrum may be reproduced reasonably well by standard TD‐DFT calculations. The only nearby state is derived from a spin‐orbit split ^2^Π_g_ state that is optically dark (g←g transitions are parity forbidden). Another interesting feature of the A state is an avoided crossing along the Au−Au coordinate with the higher‐lying G^2^Σ_u_^+^ state of the same symmetry, leading to a potential maximum of the A state near 4 Å. To spectroscopically characterize the lowest‐energy A state of Au_2_^+^, we add a weakly bonded inert argon atom as a tag. The Ar tag causes only a small perturbation of bare Au_2_^+^, because the weak van der Waals type Au_2_^+^⋅⋅⋅Ar bond (<0.5 eV)^[8]^6ek; is substantially weaker than the chemical Au−Au bond (∼2 eV).^[6]^ Thus, in addition to reducing the temperature of Au_2_^+^, the Ar tag drastically reduces the effective dissociation energy of the considered cation, thereby enabling single‐photon dissociation from the vibronic ground state.[^7^, ^8^, ^9^]

**Figure 1:**
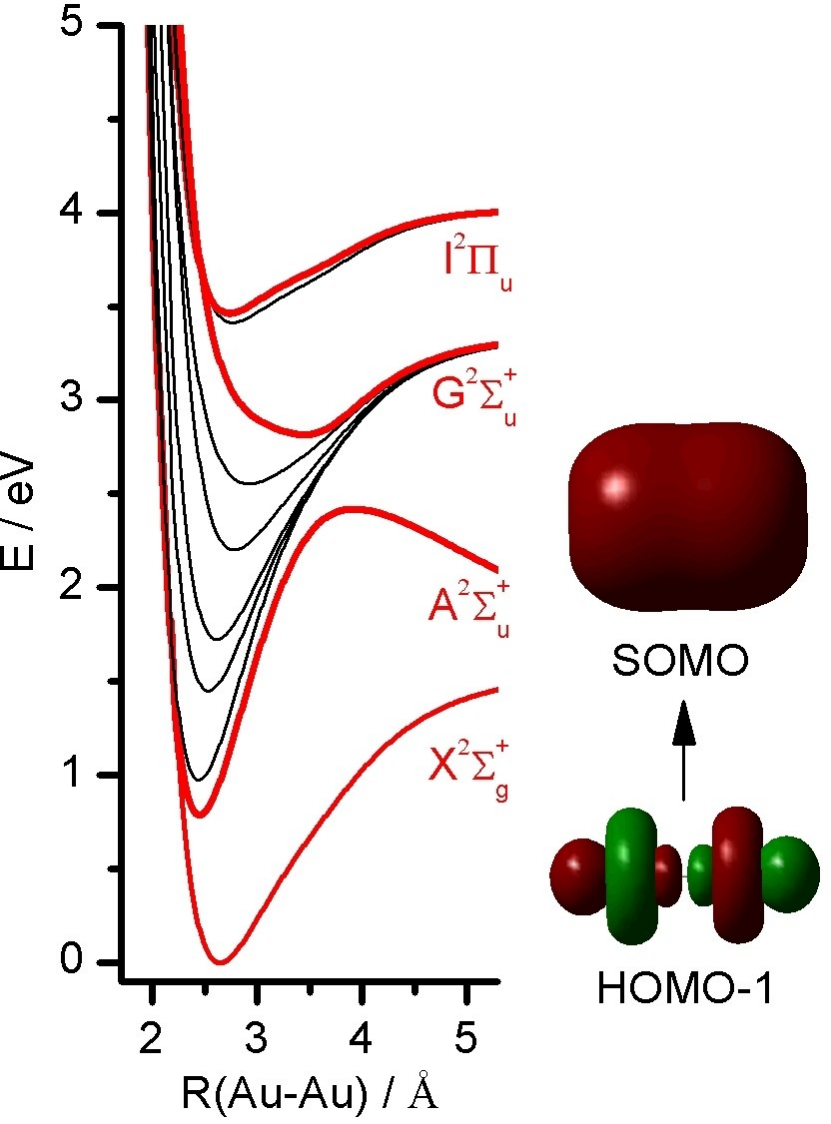
Potential energy curves of the doublet electronic states of Au_2_^+^ calculated at the unrestricted CAM‐B3LYP/def2‐QZVPP level. Optically allowed excited states are indicated in red. The avoided crossing between the G and A states of ^2^Σ_u_^+^ symmetry leads to a substantial change in the potential of both states. Without crossing, the G state would be dissociative, whereas the A state would have its dissociation limit near 3.5 eV. Also shown is the σ_u_^*^(d) antibonding HOMO‐1 from which the electron is excited into the bonding σ_g_(s) SOMO upon A←X excitation.

The NIR electronic spectrum of cold and mass‐selected Au_2_^+^Ar ions shown in Figure 2 is obtained in a QP‐ReTOF (quadrupole−reflectron time‐of‐flight) tandem mass spectrometer coupled to a temperature‐controlled pulsed laser desorption source and a broadly tunable optical parametric oscillator (OPO) laser with a bandwidth of ∼5 cm^−1^.[^3b^, ^5^] In short, Au_2_^+^Ar clusters are generated by laser vaporization of a gold rod and expanding the resulting plasma using He carrier gas seeded with Ar (0.1 %, 10 bar) into vacuum through a conical nozzle cooled by liquid nitrogen (*T*=120 K). After mass selection in the QP located behind the skimmer, the Au_2_^+^Ar ions are guided into the extraction region of the orthogonal ReTOF, where they are overlapped in space and time with the pulsed OPO laser beam. While the OPO laser operates at 10 Hz, ions are generated at a rate of 20 Hz, allowing for recording laser‐off and laser‐on spectra of parent and parent plus fragment ions, respectively. The resulting signals of Au_2_^+^Ar parent and Au_2_^+^ fragment ions are integrated and plotted as a function of laser wavelength to yield the resulting NIR spectrum, which is corrected for photon flux and overlap between ion and laser beams. No other fragment channel (e. g., Au^+^) is observed. The derived photodissociation cross section^[5]^ represents a lower limit for the optical absorption cross section because it does not account for absorption into long‐lived excited states and radiative decay (e. g., fluorescence).

**Figure 2:**
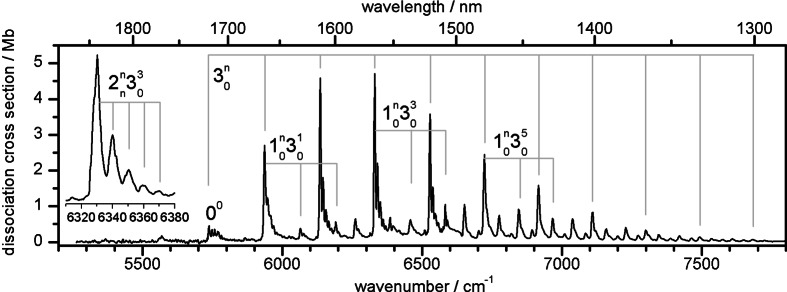
NIR photodissociation spectrum of the Ã^2^Σ^+^←$\tilde{X}$ ^2^Σ^+^ transition of Au_2_^+^Ar recorded in the Au_2_^+^ fragment channel at a nozzle temperature of 120 K. The two main progressions in the Au−Au and Au−Ar stretch modes (*ν*_3,_ *ν*_1_) are indicated. The inset shows the $2_{n}^{n}3_{0}^{3}$ progression. All peak positions and assignments are listed in Table S1.

In stark contrast to the higher excited states of Au_2_^+^ in the visible range,^[3a]^ the NIR spectrum of Au_2_^+^Ar recorded between 5000 and 8000 cm^−1^ (Figure 2) shows a regular vibronic pattern of a single excited state of a linear molecule. No other transition is observed within 5000–10 000 cm^−1^. Isolated vibronic peaks have a width of 5 cm^−1^, which corresponds to the laser bandwidth and provides a lower limit for the lifetime of *τ*=1 ps for the Ã state. The band origin (0^0^) of the Ã^2^Σ^+^←${\tilde{X}}^{2}$ Σ^+^ transition (correlating with A^2^Σ_u_^+^←X^2^Σ_g_^+^ for bare Au_2_^+^) is observed at 5738 cm^−1^ (1743 nm, 0.711 eV). A long progression of up to ten quanta in the *ν*_3_ mode with a harmonic frequency of *ω*_3_=201(1) cm^−1^ (*ν*_3_=199 cm^−1^) is assigned to the Au−Au stretch vibration, based on comparison with the higher excited states of Au_2_^+^ and the frequency of neutral Au_2_ (*ν*_3_=190 cm^−1^ in the A state).[^3a^, ^4^] The long *ν*_3_ progression peaking at *n*=3 indicates a substantial change in the Au−Au bond length upon electronic excitation. Each member of the *ν*_3_ progression is combined with a shorter progression (up to four quanta) in the *ν*_1_ mode with *ω*_1_=133(1) cm^−1^ (*ν*_1_=128 cm^−1^), which is attributed to the intermolecular Au−Ar stretch vibration. The Franck‐Condon (FC) intensities of the shorter *ν*_1_ progression suggest that the intermolecular Au_2_^+^⋅⋅⋅Ar interaction is only moderately affected by electronic excitation. In combination with the band origin and the low‐frequency members of the *ν*_1/3_ progressions and combination bands, we resolve pronounced satellite peaks with a spacing of 12 cm^−1^ and decreasing intensity (inset in Figure 2). These bands are assigned to sequence hot bands ($2_{n}^{n}$ ) in the low‐frequency degenerate Au−Au−Ar bending mode (*ν*_2_), indicating that *ν*_2_ increases by 12 cm^−1^ upon Ã←$\tilde{X}$ excitation. In contrast to *ν*_1/3_, there are no obvious intense progressions in *ν*_2_, consistent with a linear structure in both electronic states. Actually, the FC analysis described below suggests the observation of weak combination bands involving two quanta in *ν*_2_ (e. g., $2_{0}^{2}3_{0}^{4}$ and $2_{0}^{2}3_{0}^{5}$ ), resulting in *ν*_2_ =43(2) cm^−1^. From the $2_{n}^{n}$ sequence hot bands, we then derive the frequency of *ν*_2_ in the $\tilde{X}$ state as 31(2) cm^−1^. The transition at 5570 cm^−1^ occurs 169(3) cm^−1^ below the band origin. It does not fit into the regular pattern of *ν*_1/3_ and thus is assigned to the hot band in *ν*_3_ ($3_{1}^{0}$ ). Hence, the *ν*_3_ frequency increases from 169 to 199 cm^−1^ upon Ã←$\tilde{X}$ excitation, indicating a much stronger and shorter Au−Au bond in the A state. The harmonic frequencies and (cross) anharmonicities are obtained by fitting all vibronic transitions to a standard Dunham expansion (Table S1 in the Supporting Information), yielding harmonic frequencies of *ω*_1_=133.08(2) cm^−1^, *ω*_3_=200.97(2) cm^−1^, *ω*_1_ *x*_1_=−1.33(1) cm^−1^, *ω*_3_ *x*_3_=−0.56(1) cm^−1^, and *x*_13_=−1.33(1) cm^−1^ for the Ã state. As expected, the softer Au−Ar stretch mode has a larger anharmonicity than the stiffer Au−Au stretch mode. The small number of observed quanta in *ν*_2_ does not allow for a Dunham fit. A list with all experimental and fitted frequencies, along with vibrational assignments, is available in Table S1. All observed transitions can be reproduced to within 2.5 cm^−1^ with a standard deviation of 1.0 cm^−1^, which is well below the bandwidth of the employed OPO laser (5 cm^−1^).

The Birge‐Sponer (BS) plot for the long progression in *ν*_3_ (Figure 3c) yields an effective dissociation energy of *D*_0_=2.2(2) eV for the Au−Au bond in the A state, which would converge to the G state asymptote without avoided crossing (Figure 1). However, this BS approach does not account for the avoided crossing of the A excited state potential and thus provides only a safe upper limit for *D*_0_. At first glance, this result is somewhat inconsistent with the reported experimental value of *D*_0_=2.2(2) eV for the X state obtained from mass spectrometry,^[6]^ because the excited A state is certainly substantially more strongly bound than the ground state, as inferred from the increase in bond order and *ω*_3_ and the contraction of the Au−Au bond upon A←X excitation (Figure 1, Table 1). Hence, the BS analysis may suggest that the *D*_0_ value determined by mass spectrometry for the X state^[6]^ is slightly higher than the true value, as also indicated by high‐level CCSD(T) calculations (*D*_0_=1.98 eV).^[3a]^ However, the BS analysis suffers from several approximations. While Ar tagging has only a minor impact on the Au_2_^+^ potentials for the considered electronic X and A states (Table 1), the *ν*_3_ mode is not a pure Au−Au stretch local mode, because of coupling to the Au−Ar stretch, and thus corresponds only approximately to the force constant of the Au−Au bond. Finally, the BS model is based on a Morse potential (and we probe only the lowest energy part of this potential), while the true Au^+^⋅⋅⋅Au interaction at long range is dominated by charge‐induced dipole interaction (depending on *R* ^−4^).

**Figure 3:**
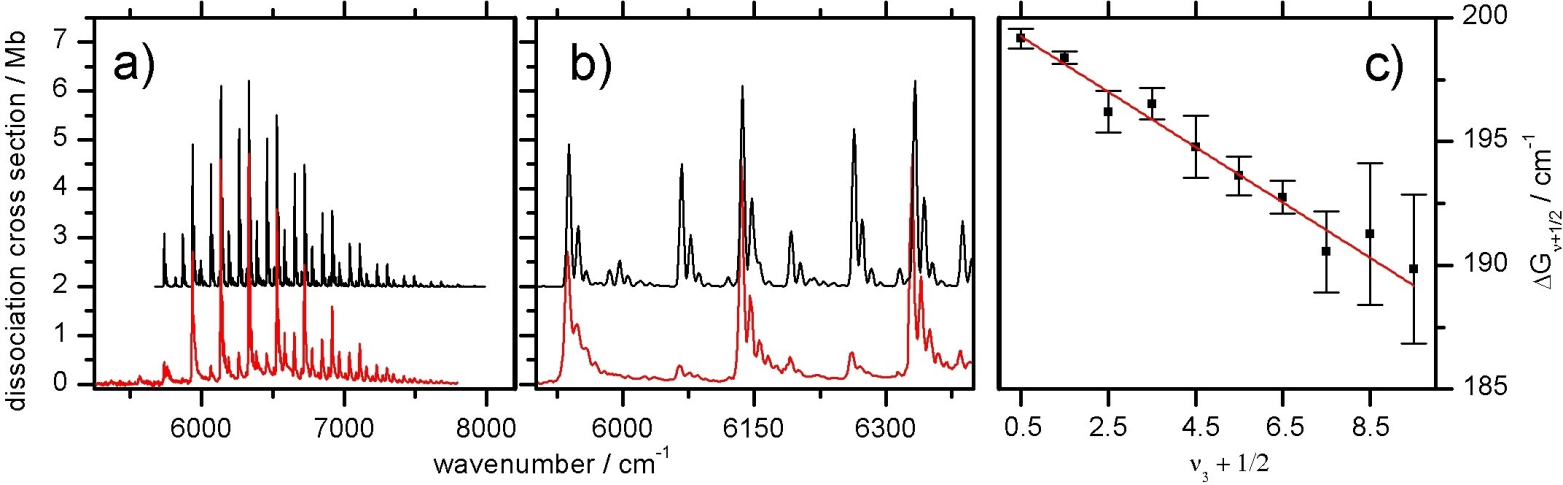
a) Photodissociation cross section of Au_2_^+^Ar into Au_2_^+^ and Ar (red) compared to FC simulations (black). Assignments are given in Figure 2. b) Expanded view of the spectra in (a). c) BS plot for the *ν*_3_ progression in the Ã^2^Σ^+^ state of Au_2_^+^Ar.

**Table 1:** Experimental properties of the ground and first excited state of Au_2_^+^ and Au_2_^+^Ar compared to computed values.

	Experiment				CAM‐B3LYP/cc‐pVTZ^[a]^				CCSD(T)^[b]^	CASSCF+MRCI+SO^[b]^	
	Au_2_Ar^+^		Au_2_^+^		Au_2_Ar^+^		Au_2_^+^		Au_2_^+^	Au_2_^+^	
State	$\tilde{X}$ ^2^Σ^+^	Ã^2^Σ^+^	X^2^Σ_g_^+^	A^2^Σ_u_^+^	$\tilde{X}$ ^2^Σ^+^	Ã^2^Σ^+^	X^2^Σ_g_^+^	A^2^Σ_u_^+^	X^2^Σ_g_^+^	X^2^Σ^+^	A^2^Σ_u_^+^
*E*_a_/eV		0.711(1)				0.79		0.77			0.95
*E*_v_/eV		0.79(3)				0.95		0.94			1.1
*R*_e_(Au−Ar)/Å	Δ(*R*‘*R*‘‘)=0.175^[c]^				2.5784	2.6927					
*r*_e_(Au−Au)/Å	Δ(*r*‘*r*‘‘)=0.09 ^[c]^				2.6226	2.4548	2.6345	2.4471	2.62	2.64	
*D*_0_(Au_2_^+^−Ar)/eV	<0.7				0.37	−0.42^[d]^					
*D*_0_(Au−Au^+^)/eV		<2.2(2)	2.2(2)^[e]^		1.94 ^[f]^		2.10	1.33	1.98	1.84	
*ω*_1_/cm^−1^		133(1)			117	131	140^[g]^	193^[g]^			
*ω*_2_/cm^−1^	31(2)^[h]^	43(2)^[h]^			31	41					
*ω*_3_/cm^−1^	169(2)^[h]^	201(1)			167	203					

[a] With ECP60MDF and GD3BJ. [b] Ref. [3a]. [c] Estimated from fitting computed to experimental relative intensities by variation of the difference in bond distances in FC simulations. [d] The potential of the A state converges asymptotically to that of the X state. The barrier between the minimum and local maximum of the A state potential of Au_2_^+^ at about *R*_e_=3.8 Å is 1.2 eV (Figure 1).^[3a]^ [e] Ref. [6]. [f] Calculated as *E*_0_(AuAr^+^)+*E*_0_(Au)−*E*_0_(Au_2_^+^Ar). [g] *ω*_1_ in Au_2_^+^ corresponds to *ω*_3_ in Au_2_Ar^+^ (Au−Au stretch). [h] Frequencies of fundamentals (*ν*_2/3_).

To confirm the assignment of the NIR spectrum, the ground and excited states of Au_2_^+^ and Au_2_^+^Ar are characterized by dispersion‐corrected (TD‐)DFT calculations at the unrestricted CAM‐B3LYP/cc‐pVTZ^[10]^ level, including GD3BJ dispersion corrections^[10b]^ and the ECP60MDF effective core potential (ECP),^[11]^ as implemented in Gaussian16.^[12]^ Calculations using the def2‐tzvpp basis yield essentially the same results (Table S2). Optimized geometries and experimentally obtained harmonic frequencies and corrections are used to fit the FC intensities of the spectrum in PGOPHER.^[13]^ To obtain the geometry differences in ground and excited state (Table 1), the atomic positions are shifted until the agreement with the experimental line intensities is optimal (Figure 3).^[13]^ The resulting differences in the geometry are given in Table 1. While the relative intensities of *ν*_1_ and *ν*_3_ can be reproduced well, the intensities of the *ν*_1_ combination bands ($1_{0}^{n}3_{0}^{m}$ ) are overestimated in the simulation for low quanta of *ν*_3_ (Figure 3b).

The properties computed for Au_2_^+^ and Au_2_^+^Ar are summarized in Table 1, along with other available computational and experimental data. The X^2^Σ_g_ ground state of Au_2_^+^ has an equilibrium bond length of *r*_e_=2.6345 Å and an Au−Au stretch frequency of 140 cm^−1^. Its dissociation energy of *D*_0_=1.94 eV is in excellent agreement with the CCSD(T) value of 1.98 eV but somewhat lower than the experimental value of *D*_0_=2.2±0.2 eV obtained from mass spectrometry^[6]^ or 2.3±0.2 eV obtained by comparing the ionization potentials of the neutral atom and dimer and the neutral dissociation energy.^[14]^ The corresponding ${\tilde{X}}^{2}$ Σ ground state of Au_2_^+^Ar is linear (*C*_∞v_) and no bent minimum is found on the potential. The intermolecular Au−Ar bond is characterized by *R*_e_=2.578 Å and *D*_0_=0.37 eV, and the intermolecular harmonic stretch and bend frequencies are *ω*_1_=117 cm^−1^ and *ω*_2_=31 cm^−1^. The much stronger intramolecular Au−Au bond is characterized by *r*_e_=2.6226 Å and *D*_0_=1.94 eV, with a harmonic stretch frequency of *ω*_3_=167 cm^−1^, in excellent agreement with the measured value of *ν*_3_=169 cm^−1^ derived from the hot band in the NIR spectrum. Ar tagging has only a minor stabilizing effect on the Au−Au bond (Δ*r*_e_=−11.9 mÅ), probably arising from partial electron transfer (0.1 *e*) from Ar into the bonding SOMO of Au_2_^+^. Because of strong coupling between the Au−Au and Au−Ar local modes in the $\tilde{X}$ (Au−Au stretch) corresponds to *ω*_1_ in Au_2_^+^).

The first excited state of Au_2_^+^ is the optically bright A state (^2^Σ_u_^+^) with a predicted adiabatic transition energy of *E*_a_=0.77 eV and relatively low oscillator strength (ƒ=0.009). It arises from 6s←5d excitation of an electron out of the antibonding σ_u_^*^ orbital (HOMO‐1) into the bonding σ_g_ orbital (SOMO). As a consequence of the increase in bond order, the calculated Au−Au bond contracts substantially by Δ*r*_e_=−187 mÅ and the Au−Au stretch frequency increases from 140 to 193 cm^−1^. Because of the drastic change in geometry, there is a huge difference of 30 % between adiabatic and vertical transition energy (*E*_a/v_=0.94/0.77 eV), indicating that reliable predictions for transition energies require optimization of the excited state. Most previous computations of Au_*n*_^+^ clusters rely merely on the calculation of vertical electronic excitations.[^6^, ^7^, ^15^] In our particular case, the predicted NIR transition shifts from 1320 to 1610 nm upon geometry optimization. Ar complexation increases the oscillator strength to *f*=0.0017 and has only a minor stabilizing impact on the Au−Au bond in the Ã state (Δ*r*_e_=−7.7 mÅ, Δ*ω*_2_=+12 cm^−1^). Overall, Ã←$\tilde{X}$ excitation of Au_2_^+^Ar leads to a substantial contraction of the strong Au−Au bond (Δ*r*_e_=−168 mÅ) and a similar elongation of the soft Au−Ar bond (Δ*R*_e_=114 mÅ). While the strengthening of the Au−Au bond is well reflected by the increase in *ω*_3_ from 167 to 203 cm^−1^, the weakened Au−Ar bond also exhibits an increase in *ω*_1_ from 117 to 131 cm^−1^. This at first glance inconsistent picture arises from the change in coupling between the Au−Ar and Au−Au local modes upon Ã←$\tilde{X}$ excitation. While both modes are strongly coupled in the $\tilde{X}$ state leading to an enhanced splitting between both normal modes, they become almost decoupled in the Ã state. The frequency of the degenerate intermolecular bending mode *ω*_2_ increases from 31 to 43 cm^−1^ (or 40 %), indicating a much stiffer bending potential in the Ã state, with higher angular anisotropy.

Overall, the (TD‐)DFT calculations reproduce the experimental observation to high accuracy. The assigned band origin at 5738 cm^−1^ (0.711(2) eV) agrees well with the predicted value (0.79 eV). Ar complexation changes *E*_a_ and *E*_0_ of Au_2_^+^ by only 0.02 and 0.01 eV, respectively, thus confirming that the Ar tag has essentially no effect on this electronic transition. The measured frequencies in the Ã state (*ω*_1/3_=133/201 cm^−1^) agree with the computed ones (131/203 cm^−1^) to within 2 cm^−1^. The computed increase of 10 cm^−1^ in *ω*_2_ upon Ã←$\tilde{X}$ excitation is close to the observed spacing of 12 cm^−1^ in the $2_{n}^{n}$ sequence hot band progression. Similarly, the *ν*_3_ frequency in the $\tilde{X}$ state (169 cm^−1^) assigned from the hot band is consistent with the predicted value of *ω*_3_=167 cm^−1^. This overall excellent quantitative match between experiment and computation with respect to electronic transition energy and all vibrational frequencies illustrates that the employed standard (TD‐)DFT calculations are surprisingly well suited to reliably describe the chemical bonding and electronic structure of the X and A state of Au_2_^+^, and is rationalized by the, at most, small perturbation of these isolated electronic states (Figure 1). This scenario is not true anymore for the higher excited states, which are strongly coupled.^[3a]^

The relative intensities of the hot band transitions may be used to estimate the effective vibrational temperature of the cluster ions. Assuming thermal equilibrium (Boltzmann distribution) and similar FC factors for the hot band and fundamental of *ν*_3_ ($3_{1}^{0}$ and $3_{0}^{1}$ ), their observed intensity ratio of 1 : 10 reflects directly the population ratio of the *ν*_3_ and ground states. This ratio corresponds to a temperature of around 110 K, which agrees well with the nozzle temperature of *T*=120 K. The ion temperature can also be estimated from the population of the *ν*_2_ levels in the $\tilde{X}$ state derived from the observed sequence hot bands spaced by 12 cm^−1^, again assuming the same FC factors. For example, the intensity ratio of $3_{0}^{n}$ :$3_{0}^{n}2_{1}^{1}$ :$3_{0}^{n}2_{2}^{2}$ with *n*=1–3 is within 4.8 : 1.8 : 1.0 and 1.5 : 1.2 : 0.9, and varies between different scans with equal source conditions but also within single scans. Again, assuming a Boltzmann distribution, these ratios translate into *T*=50–200 K, respectively. The FC simulations show a slightly different picture. To achieve good agreement in the hot band contributions, we need to consider a different temperature for *ν*_2_ compared to *ν*_1_ and *ν*_3_. The best fit is obtained with a *ν*_2_ temperature of around 25 K, while *ν*_1_ and *ν*_3_ have an effective temperature of around 200 K. This result is not surprising because low‐frequency modes cool more efficiently than high‐frequency vibrations.

In summary, we have characterized the previously elusive first electronically excited A state of the fundamental Au_2_^+^ diatomic cation by means of high‐resolution photodissociation spectroscopy of the Ar‐tagged ion. Significantly, the measured A←X spectrum provides the first spectroscopic information about the chemical bonding of Au_2_^+^ in both the ground and first excited state. The exceptionally low‐lying excited A state occurring in the NIR range is dominated by a long vibrational progression in the Au−Au stretch mode caused by a substantial bond contraction upon electronic excitation. This change in geometry is caused by one‐electron excitation from an antibonding σ_u_^*^ orbital into the bonding σ_g_ orbital. The vibronically resolved spectrum allows for the determination of all three vibrational modes in the Ã state of Au_2_^+^Ar and of two frequencies in the $\tilde{X}$ state via hot band analysis. Analysis of anharmonicity provides a safe upper limit of the dissociation energy in the A state as 2.2(2) eV. The true dissociation energy should be substantially lower because the BS analysis does not account for the effects of the avoided crossing of the A state with a higher lying excited state. Significantly, the TD‐DFT calculations describe the properties of the largely isolated X and A state to surprisingly high accuracy (as seen for example also by the negligible spin contamination of <2 %), given that an excited state of such a heavy diatomic open‐shell cation is considered and that the properties of higher excited states cannot be reproduced at such level of theory.^[3a]^ Comparison between Au_2_^+^ and Au_2_^+^Ar reveals that rare gas tagging has essentially no impact on the geometric and electronic structure of the diatomic cation, while electronic excitation reduces the coupling between the Au−Au and Au−Ar stretch modes.

Recent computations predict that open‐shell Au_*n*_^+^ clusters with even *n* (*n*≤12) have low‐energy electronic states in the NIR range. The most extreme case in this size range is Au_10_^+^, for which the onset of a rather broad and unresolved band is observed (also by Ar tagging), with a fitted maximum near 5000 cm^−1^ (∼0.6 eV, ∼2000 nm) and a width of ∼4400 cm^−1^ (∼0.55 eV). This band has been attributed to three overlapping LUMO←SOMO electronic transitions, and its large width has been rationalized by spectral congestion from unresolved vibronic excitation, vibronic coupling of the Jahn‐Teller distorted tetrahedral structure, and/or lifetime broadening.^[7]^ In contrast to the larger and more complex Au_10_^+^ cluster, the lowest‐energy NIR excitation of Au_2_^+^ observed at 0.71 eV arises from a single and well‐isolated SOMO←HOMO‐1 transition resulting in a regular well‐resolved electronic spectrum, with a long‐lived excited state (*τ*≥1 ps). Significantly, the Au_2_^+^Ar spectrum allows the determination of all vibrational frequencies, thereby providing very detailed information about the Au−Au and Au−Ar bonds as a function of electronic excitation. Such highly resolved electronic spectra are still rare for transition metal clusters.[^3^, ^8a^, ^16^]

## Conflict of interest

The authors declare no conflict of interest.

## Supporting information

As a service to our authors and readers, this journal provides supporting information supplied by the authors. Such materials are peer reviewed and may be re‐organized for online delivery, but are not copy‐edited or typeset. Technical support issues arising from supporting information (other than missing files) should be addressed to the authors.

Supporting Information
