# Supplementary material for: Near‐Infrared Spectrum of the First Excited State of Au2 +
Source: Chemistry. 2021 Sep 15;27(61):15075–80. doi: 10.1002/chem.202102542 (PMC8596823; doi:10.1002/chem.202102542)
Supplement: Supplementary file 1 — Supporting Information [file CHEM-27-15075-s001.pdf]

Chemistry–A European Journal

Supporting Information

Near-Infrared Spectrum of the First Excited State of Au_2^+

Marko Förstel,* Kai Pollow, Taarna Studemund, and Otto Dopfer*

Table of Content for Supplementary Supporting Information

Table S1. List of experimental and fitted line positions for the $\tilde{A}^2\Sigma^+ \leftarrow \tilde{X}^2\Sigma^+$ transition of Au_2^+Ar.

Table S2. Comparison of selected parameters of Au_2^+ and Au_2^+Ar obtained with two different (TD-)DFT levels of theory.

Figure S1. Relevant Au_2^+ and Au_2^+Ar molecular orbitals.

Table S1. List of absolute and relative (Δ) experimental line positions and vibrational assignments (n_1, n_2, n_3) for the $\tilde{A}^2\Sigma^+ \leftarrow \tilde{X}^2\Sigma^+$ transition of Au_2^+Ar compared to calculated frequencies (all values in cm^{-1}) resulting from a fit to the following Dunham expansion:

$$E(n_1, n_3) = E(0,0) + \omega_3 n_3 + \omega_1 n_1 + \omega_3 x_3 (n_3{}^2 + n_3) + \omega_1 x_1 (n_1{}^2 + n_1) + x_{13}\left(n_3 n_1 + \frac{n_3}{2} + \frac{n_1}{2}\right)$$

with ω_1=133.08(2) cm^{-1}, ω_3=200.97(2) cm^{-1}, $\omega_1 x_1$=-1.33(1) cm^{-1}, $\omega_3 x_3$=-0.56(1) cm^{-1}, and x_{13}=-1.33(1) cm^{-1}.[a]

$\tilde{A} \leftarrow \tilde{X}$ Transition	Experiment[b]	Δ[c]	Calculation	Δ[c]
(0, 0, 0) ← (0, 0, 0)	5737.56	0.00	0.00	0.00
(0, 0, 1) ← (0, 0, 0)	5936.48	198.92	199.19	0.28
(0, 0, 2) ← (0, 0, 0)	6134.97	397.41	397.27	-0.13
(0, 0, 3) ← (0, 0, 0)	6331.12	593.55	594.24	0.69
(0, 0, 4) ← (0, 0, 0)	6527.84	790.28	790.10	-0.18
(0, 0, 5) ← (0, 0, 0)	6722.24	984.67	984.85	0.18
(0, 0, 6) ← (0, 0, 0)	6916.11	1178.54	1178.49	-0.06
(0, 0, 7) ← (0, 0, 0)	7108.84	1371.27	1371.01	-0.26
(0, 0, 8) ← (0, 0, 0)	7299.27	1561.71	1562.43	0.72
(0, 0, 9) ← (0, 0, 0)	7490.64	1753.07	1752.73	-0.34
(0, 0, 10) ← (0, 0, 0)	7680.49	1942.93	1941.93	-1.00
(1, 0, 0) ← (0, 0, 0)	5865.10	127.54	129.76	2.22
(1, 0, 1) ← (0, 0, 0)	6064.28	326.72	327.62	0.90
(1, 0, 2) ← (0, 0, 0)	6260.76	523.20	524.37	1.18
(1, 0, 3) ← (0, 0, 0)	6457.86	720.30	720.01	-0.29
(1, 0, 4) ← (0, 0, 0)	6651.15	913.58	914.54	0.96
(1, 0, 5) ← (0, 0, 0)	6846.97	1109.41	1107.96	-1.45
(1, 0, 6) ← (0, 0, 0)	7037.30	1299.73	1300.26	0.53
(1, 0, 7) ← (0, 0, 0)	7228.04	1490.48	1491.46	0.98
(1, 0, 8) ← (0, 0, 0)	7418.40	1680.83	1681.54	0.71
(1, 0, 9) ← (0, 0, 0)	7607.46	1869.89	1870.52	0.63
(2, 0, 1) ← (0, 0, 0)	6190.03	452.47	453.40	0.93
(2, 0, 2) ← (0, 0, 0)	6385.70	648.13	648.82	0.68
(2, 0, 3) ← (0, 0, 0)	6583.28	845.71	843.12	-2.59
(2, 0, 4) ← (0, 0, 0)	6775.07	1037.50	1036.32	-1.18
(2, 0, 5) ← (0, 0, 0)	6966.21	1228.65	1228.41	-0.24
(2, 0, 6) ← (0, 0, 0)	7158.20	1420.63	1419.38	-1.25
(2, 0, 7) ← (0, 0, 0)	7344.84	1607.28	1609.25	1.97
(2, 0, 8) ← (0, 0, 0)	7535.80	1798.23	1798.00	-0.23
(3, 0, 1) ← (0, 0, 0)	6315.12	577.56	576.51	-1.05
(3, 0, 2) ← (0, 0, 0)	6508.30	770.73	770.60	-0.13
(3, 0, 3) ← (0, 0, 0)	6702.41	964.85	963.58	-1.27
(3, 0, 4) ← (0, 0, 0)	6891.80	1154.23	1155.45	1.21
(3, 0, 5) ← (0, 0, 0)	7084.66	1347.10	1346.20	-0.90
(3, 0, 6) ← (0, 0, 0)	7272.73	1535.16	1535.85	0.68

(3, 0, 7) ← (0, 0, 0)	7462.69	1725.12	1724.38	-0.74
(3, 0, 8) ← (0, 0, 0)	7651.11	1913.55	1911.81	-1.74
(4, 0, 2) ← (0, 0, 0)	6626.91	889.34	889.73	0.39
(4, 0, 3) ← (0, 0, 0)	6818.96	1081.39	1081.38	-0.02
(4, 0, 4) ← (0, 0, 0)	7008.94	1271.37	1271.91	0.54
(4, 0, 5) ← (0, 0, 0)	7199.42	1461.86	1461.34	-0.52
(4, 0, 6) ← (0, 0, 0)	7386.89	1649.32	1649.65	0.33
(4, 0, 7) ← (0, 0, 0)	7572.89	1835.33	1836.86	1.53
(0, 2, 4) ← (0, 0, 0)	6613.76	876.19		
(0, 2, 5) ← (0, 0, 0)	6807.35	1069.79		
(0, 1, 0) ← (0, 1, 0)	5749.17	11.61		
(0, 1, 1) ← (0, 1, 0)	5947.47	209.90		
(0, 1, 2) ← (0, 1, 0)	6145.19	407.63		
(0, 1, 3) ← (0, 1, 0)	6341.20	603.63		
(0, 1, 4) ← (0, 1, 0)	6538.08	800.52		
(0, 1, 5) ← (0, 1, 0)	6731.74	994.18		
(0, 1, 6) ← (0, 1, 0)	6925.21	1187.64		
(0, 1, 7) ← (0, 1, 0)	7117.44	1379.87		
(0, 1, 8) ← (0, 1, 0)	7307.27	1569.71		
(0, 2, 0) ← (0, 2, 0)	5760.37	22.80		
(0, 2, 1) ← (0, 2, 0)	5957.70	220.14		
(0, 2, 2) ← (0, 2, 0)	6154.60	417.04		
(0, 2, 3) ← (0, 2, 0)	6353.24	615.68		
(0, 2, 4) ← (0, 2, 0)	6544.50	806.94		

[a] uncertainties of the values are obtained from fit using all tabularized line positions.
[b] absolute systematic error in line positions is 5 cm^{-1} arising from the laser bandwidth and the error in the calibration in the laser wavelength.
[c] deviation from the experimental value.

Table S2. Comparison of selected parameters obtained with two different (TD-)DFT levels of theory (compare Table 1 in the main manuscript).

	CAM-B3LYP/def2-tzvpp				CAM-B3LYP/cc-pVTZ			
	Au_2Ar^+		Au_2^+		Au_2Ar^+		Au_2^+	
state	$\tilde{X}^2\Sigma^+$	$\tilde{A}^2\Sigma^+$	$X^2\Sigma_g^+$	$A^2\Sigma_u^+$	$\tilde{X}^2\Sigma^+$	$\tilde{A}^2\Sigma^+$	$X^2\Sigma_g^+$	$A^2\Sigma_u^+$
E_a / eV		0.8320		0.8043		0.79		0.77
E_v / eV		1.0005		0.9721		0.95		0.94
R_e(Au-Ar) / Å	2.5617	2.6781			2.5784	2.6927		
r_e(Au-Au) / Å	2.6224	2.4513	2.9396	2.4520	2.6226	2.4548	2.6345	2.4471
D_0(Au_2^+-Ar) / eV	0.3879	-0.44			0.37	-0.42		
D_0(Au-Au^+) / eV	1.89		2.0328	1.2285	1.94		2.10	1.33
ω_1 / cm^{-1}	119	133	138	191	117	131	140	193
ω_2 / cm^{-1}	33	42			31	41		
ω_3 / cm^{-1}	170	203			167	203		

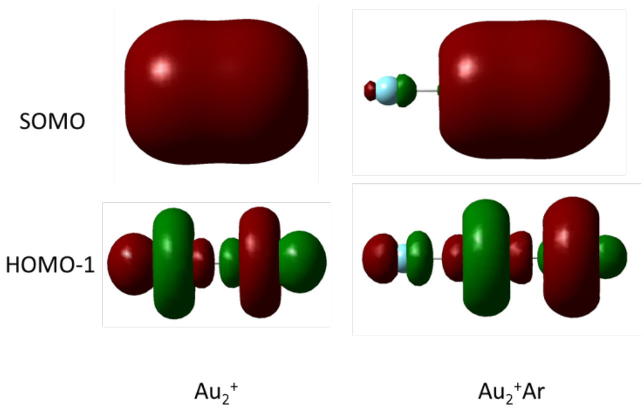

Figure S1. Comparison of the relevant Au_2^+ and Au_2^+Ar molecular orbitals. An electron is moved from a nonbonding $\sigma_u^*(d)$ orbital (HOMO-1) to the bonding $\sigma_g(s)$ orbital (SOMO).
